# Perfect people, happier lives? When the quest for perfection compromises happiness: the roles played by substance use and internet addiction

**DOI:** 10.3389/fpubh.2023.1234164

**Published:** 2023-09-28

**Authors:** Alexandra Maftei, Cristian Opariuc-Dan

**Affiliations:** Faculty of Psychology and Education Sciences, Alexandru Ioan Cuza University, Iaşi, Romania

**Keywords:** happiness, youth, perfectionism, addiction, substance use

## Abstract

Building on the Conservation of Resources Theory and the Stress-Coping Model, the present study explored the relationship between perfectionism (rigid, self-critical, narcissistic) and subjective happiness among youth. In this relationship, we also examined the mediating roles of substance use (i.e., drinking and smoking) and Internet addiction symptoms. Our sample comprised 431 Romanian university students aged 18–25 (*M* = 20.50, *SD* = 1.58), and most of them were females (79.81%, self-reported gender). Participants completed self-reported anonymous scales through a web-based survey at the beginning of 2023. Correlation analysis results indicated that all forms of perfectionism were associated with Internet addiction symptoms. Self-critical and narcissistic perfectionism and drinking, smoking, and Internet addiction symptoms were negatively associated with subjective happiness. Path analysis suggested that health-risk behaviors completely mediated the effect of perfectionism on subjective happiness. High levels of perfectionism were associated with high levels of health-risk behaviors, and high levels of addictive behaviors were associated with low levels of subjective happiness. We discuss the present findings considering their practical use regarding students' subjective happiness.

## Introduction

Over the past few decades, with the growth of positive psychology, an increasing number of studies focused on subjective happiness (or subjective wellbeing) (1). Subjective happiness refers to individuals' sense of good and wellness, including satisfaction with life, enjoyment, love, and overall positive feelings. Also, higher subjective happiness means lower distress and negative emotions, such as anger or fear (2). The reasons behind this interest could not be more important: compared with less happy people, happy individuals have better interpersonal relationships (3), are more productive (4), more socially responsible (5), have a higher academic achievement (6), and, generally, they live longer and have healthier lives (7).

However, recent estimates suggested that psychological distress (which includes feelings of depression, anxiety, and stress) (8)—as a measure of low subjective happiness, is one of the primary factors associated with reports of suicide ideation and attempts (9). Thus, it is all the more important to identify the risk factors contributing to lower happiness, especially among one particularly vulnerable population regarding this matter, i.e., university students (10). Therefore, in the present study, we focused on one less investigated factor when discussing subjective happiness, i.e., multidimensional perfectionism. Furthermore, we aimed to examine how different forms of perfectionism might indirectly affect youth's happiness through substance use (i.e., alcohol and nicotine) and Internet addiction symptoms, especially since a growing number of studies highlighted the high prevalence of co-occurrence of alcohol and tobacco use among students (11), as well as the high numbers regarding Internet addiction symptoms (12, 13).

### Not all perfectionists are the same

Recent data estimates that perfectionism is increasing over time due to cultural changes reflected in competitive individualism, highlighting that today's youth have higher expectations of themselves, their peers, and society (14). Perfectionism is considered a multidimensional personality trait, relatively stable across time (15). It is characterized by excessively high expectations of one's abilities and those around them, harsh self- and social criticism, and an obsession with perfection (16). There are various models and measures of perfectionism (17), highlighting different facets such as (a) self-oriented perfectionism, other-oriented perfectionism, and socially prescribed perfectionism (15), (b) rigid, self-critical, and narcissistic perfectionism (18), or (c) adaptive and maladaptive perfectionism (19, 20). In the present study, we investigated perfectionism through the lenses proposed by the conceptualization proposed by Smith et al. (18).

*Rigid* perfectionists expect nothing less than excellence from oneself, comprising elements of self-oriented perfectionism and reliance on external validation (18). Furthermore, rigid perfectionism describes the importance of pursuing excellence and the connection between one's perceived worth and meeting personal standards of perfection (21). Next, concerns, adverse reactions to defective or faulty performance, and the belief that others want one to be flawless are characteristics of *self-critical perfectionism* (22). Individuals high in self-critical perfectionism generally have overly adverse reactions to their perceived mistakes and failures; they experience pervasive uncertainty and dissatisfaction with their performance—since they tend to believe others demand perfection—and are overly self-critical of their perceived absence of perfection (16, 22). Finally, *narcissistic perfectionism* defines the tendency to be excessively critical of others and self-centered in one's expectations of them (18). Individuals high in narcissistic perfectionism are usually intolerant to others' mistakes, might expect special treatment due to feelings of self-entitlement, and believe they are superior to others regarding their perfection (23, 24).

### Perfectionism and substance use

According to the Conservation of Resources (COR) theory, as people's supplies run out, they may experience emotional distress, affecting their general wellbeing (25). Applying the COR theory to university students, Nelsen et al. (26) argued that students high in perfectionism may pursue excellence and high personal standards much more and consume more resources, such as energy and time, to achieve academic success. When these resources are drained over time, the emotional distress experienced by perfectionist students might be much higher than those lower in perfectionism. Furthermore, according to the stress-coping model (27), using alcohol and tobacco might be the reflection of a coping mechanism with negative emotions and to enhance positive emotions (28). Therefore, as perfectionists are less likely to lower their standards and more likely to resort to harmful coping mechanisms (29), they might also be more prone to substance use.

Some previous studies have explored the link between different facets of perfectionism and substance use. For example, some suggested that adaptive perfectionism might be related to better mental health among college students, compared to maladaptive forms of perfectionism and lower substance use (i.e., drinking and smoking) (26). Other studies suggested that maladaptive perfectionism and negative coping styles predicted substance abuse and addiction (30). Similar findings highlighted the significant link between maladaptive perfectionism and substance abuse as a coping mechanism [e.g., Rice and Van Arsdale (31)]. However, there is also mixed evidence regarding the link between perfectionism and drinking (32), highlighting the need for further evidence.

Previous studies suggested that rigid perfectionism positively predicts anxiety and negative emotions, such as guilt (14). Self-critical perfectionism is also considered a vulnerability toward stress perception, significantly predicting depression, anxiety, and risky health-related behaviors, such as eating disorders (33, 34). Since negative emotions and psychological distress are negative predictors of subjective happiness (35), we can consider the indirect association between perfectionism and happiness. Also, the positive link between psychological distress and substance use, highlighted in previous studies (36), might underline the direct relation between perfectionism and substance use proneness (37). Finally, narcissistic perfectionism, which involves being critical, demanding, arrogant, and entitled, often involves conflict and derogation (38). In response to these conflicts, individuals might use drinking and smoking as coping (maladaptive) strategies to manage the related emotional negativity.

### Perfectionism and internet addiction

Though it has many conceptualizations, Internet addiction generally describes a maladaptive form of Internet use, a psychological dependence on the Internet, characterized by symptoms of withdrawal, anxiety, and a loss of impulse control concerning the urge to use the Internet (12). According to recent estimates, generalized Internet addiction prevalence rates seem to vary from 12.6 to 67.5%, with significantly higher numbers among adolescents and university students (39). Internet addiction has many adverse outcomes regarding one's psychological and physical health, including depression, anxiety, stress symptoms, disordered sleep and eating habits (40, 41), in addition to generally lower subjective happiness (42, 43). Furthermore, among university students, Internet addiction is also predicted by substance use and abuse, such as drinking and smoking (44).

Previous studies suggested that maladaptive perfectionism is significantly associated with Internet addiction through the mediating role of depression (45). Other studies suggested that a perfectionistic attitude predicts Internet addiction even when depression is controlled (46). Socially prescribed perfectionism, a facet of self-critical perfectionism, was also significantly related to problematic Internet use in previous studies (47, 48). Next, perfectionistic concerns seem significantly related to mobile phone addiction (49) and maladaptive cognitions about the self, translated by not meeting perfectionism standards, further predicting problematic social media use (which is a form of maladaptive Internet use) (50).

Finally, building on the stress-coping model (27), we consider Internet addiction as a result of maladaptive coping when managing the emotional burden of perfectionism. Previous studies that examined the risk factors of problematic Internet use, for instance, highlighted its role as a (maladaptive) mood regulation mechanism (51). Thus, when individuals' stress is high (due to not meeting the standards of perfectionism), Internet use might become maladaptive, i.e., addictive. Consequently, we believe that high perfectionists are more prone to develop Internet addiction, which, in turn, might lead to lower subjective happiness (52–54).

### Perfect people, happier lives? The aims of the present study

Finally, we come to the question: *perfect people, happier people?* In other words, what is the connection between rigid, self-critical, and narcissistic perfectionism and subjective happiness? Previous studies suggested that perfectionism involves a rigid evaluation of daily problems and that cognitive reappraisal might reduce the unfavorable link between perfectionism and cognitive flexibility, except among individuals with strong narcissistic perfectionism (29). Since individuals high in cognitive flexibility are usually happier (55), perfectionism might indirectly affect happiness due to its negative association with cognitive flexibility. Other studies suggested that the link between various forms of perfectionism and subjective happiness is generally significant and negative (56).

Building on the COR theory (25), we believe that individuals high in perfectionism might use more resources and energy to fulfill their overly high standards than those low in perfectionism. Furthermore, building on the stress-coping model (27), due to the pressure of achieving perfection, they might also experience more emotional distress, which might make them more prone to drink, smoke, and use the Internet in a maladaptive way to cope with the perceived stress. Subsequently, these specific risky behaviors might be reflected in lower subjective happiness.

Previous studies examined the mediating role of risky behaviors on the link between perfectionism and students' mental health (26), but our research has some specific novelties. First, we conceptualized perfectionism using Smith et al.'s (18) framework, measuring rigid, self-critical, and narcissistic perfectionism. Second, we investigated not only substance abuse but also Internet addiction symptoms as an additional maladaptive coping mechanism with the potential stress caused by perfectionism. Finally, we measured subjective happiness, a less investigated construct when discussing the link with university students' rigid, self-critical, and narcissistic perfectionism.

Based on the COR theory (25), the stress-coping model (27), and previous findings examining the complex relationships between our variables of interest, our assumptions were the following:

**H**_**1**_. Perfectionism would be positively associated with health-risk behaviors, i.e., smoking, drinking, and Internet addiction symptoms.
**H**_**2**_. Health-risk behaviors would be related to lower subjective happiness.
**H**_**3**_. Health-risk behaviors would mediate the link between perfectionism and subjective happiness.

**Figure 1:**
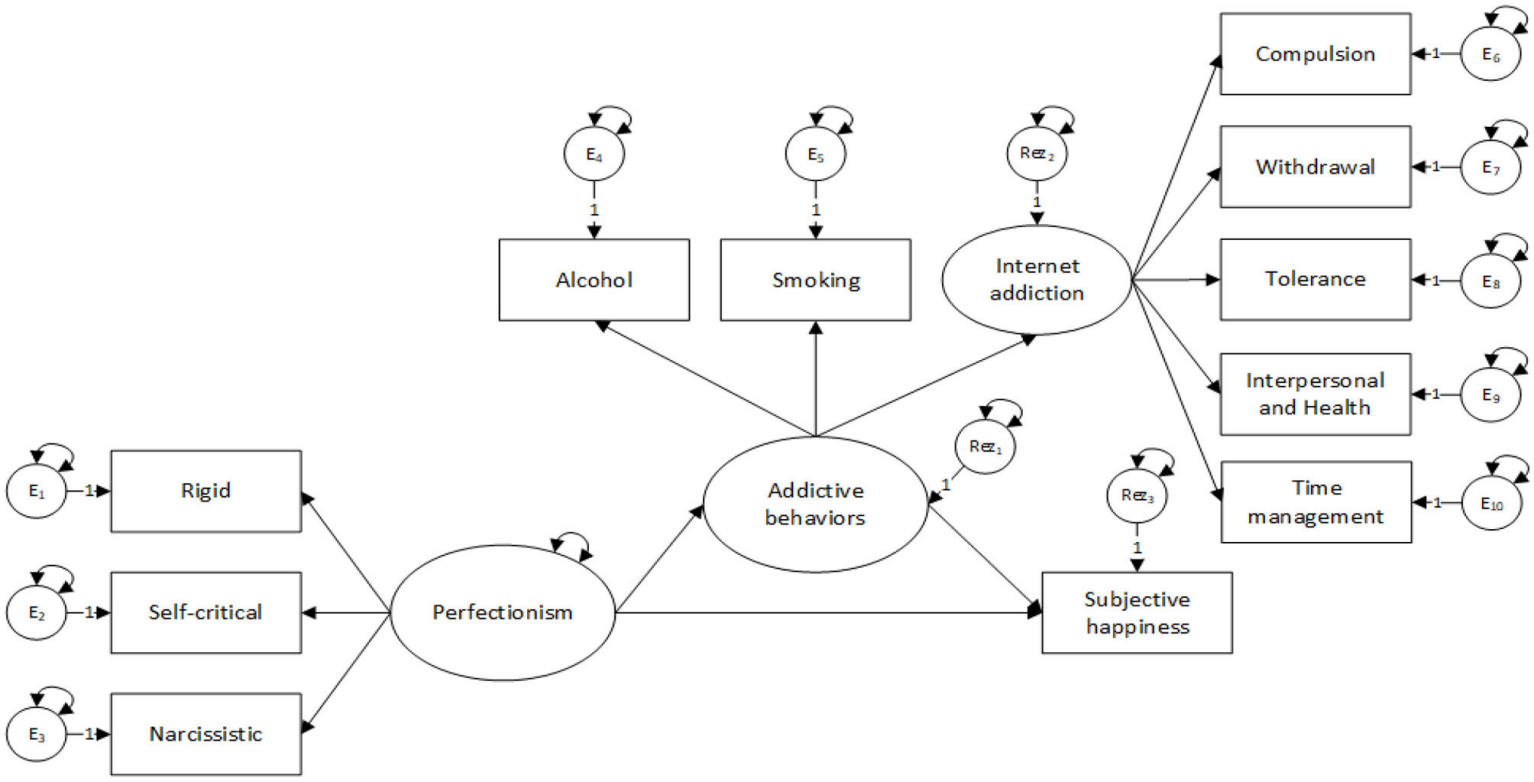
The proposed research model.

The proposed research model is detailed in Figure 1.

## Method

### Participants and procedure

Our final sample comprised 431 students from two major universities in Romania. Their ages varied from 18 to 25 (*M* = 20.50, *SD* = 1.58), and most of them were females (79.81%, self-reported gender). Using convenience sampling (i.e., snowball sampling), data were collected in January 2023 using an online form distributed through student social media groups (e.g., Facebook). The participants replied to an invitation to participate in a study project addressing the psychological issues of the digital age in exchange for course credits.

All participants provided informed consent to participate in this study. Students were advised that their participation was entirely voluntary and that they might withdraw at any time. In addition, they were told that their answers would remain anonymous and private and would be utilized just for the current research. Twelve minutes was the average time necessary to answer all questions. The research was conducted following the ethical standards established by the 2013 Helsinki Declaration and was authorized by the Ethics Committee where the authors are affiliated.

### Measures

#### Subjective happiness

We used the four-item Subjective Happiness scale developed by Lyubomirsky and Lepper (57). Example items included *Some people are generally very happy., They enjoy life regardless of what is happening, getting the most out of everything*., and *To what extent does this characterization describe you?* Participants answered the items on a 7-point Likert scale ranging from 1 (not at all) to 7 (a great deal). Higher scores indicated a higher level of subjective happiness. In the present study, Cronbach's *alpha*−*s* 0.79, 95% CI (0.75, 0.82).

#### Perfectionism

We used The Big Three Perfectionism Scale–Short Form (BTPS-SF) developed by Feher et al. (16) to measure three facets of perfectionism, i.e., *rigid* (four items, e.g., *I have a strong need to be perfect*), *self-critical* (six items, e.g., *The idea of making a mistake frightens me*), and *narcissistic* perfectionism (six items, e.g., *It bothers me when people don't notice how perfect I am*). Participants answered the items on a 5-point Likert scale ranging from 1 (disagree strongly) to 5 (agree strongly). Higher scores indicated higher perfectionism on all dimensions. In the present study, Cronbach's α for rigid perfectionism was 0.91, 95% CI (0.9, 0.92), for self-critical perfectionism was 0.88, 95% CI (0.86, 0.9), and for narcissistic perfectionism was 0.86, 95% CI (0.84, 0.88). A CFA was performed, resulting in a marginal acceptable fitted model under the orthogonal assumptions (χ^2^ = 562.72, df = 101, *p* < 0.001, TLI = 0.99, SRMR = 0.08, RMSEA = 0.10, *p* < 0.001, 90% CI (0.09, 0.11)].

#### Substance use

We measured participants' alcohol and nicotine use using the items from The Personal Experience Screening Questionnaire (PESQ) developed by Winters (58). Six items measured alcohol use (e.g., *How often have you used alcohol at home?* and *How often have you used alcohol secretly, so nobody would know you were using?*), and six items measured nicotine use, i.e., smoking (e.g., *How often have you smoked at home?* and *How often have you smoked secretly, so nobody would know you were using?*). Participants answered using a 4-point Likert scale ranging from 1 (never) to 4 (often). Higher scores indicated higher substance use. In the present study, Cronbach's α for alcohol use was 0.83, 95% CI (0.8, 0.85), and nicotine use was 0.88, 95% CI (0.87, 0.9). A CFA was also performed, resulting in a marginally acceptable fitted model under the orthogonal assumptions [χ^2^ = 325.98, df = 53, *p* < 0.001, TLI = 0.98, SRMR = 0.11, RMSEA = 0.11, *p* < 0.001, 90% CI (0.10, 0.12)].

#### Internet addiction

We used the 26-item Chen Internet Addiction (CIAN) scale developed by Chen et al. (59) to measure four dimensions of Internet addiction, i.e., *compulsion symptoms* [Cronbach's α = 0.84, 95% CI (0.81, 0.86)], *withdrawal symptoms* [Cronbach's α = 0.86, 95% CI (0.83, 0.88)], *tolerance symptoms* [Cronbach's α = 0.78, 95% CI (0.75, 0.81)], *interpersonal and health-related problems* [Cronbach's α = 0.86, 95% CI (0.84, 0.88)], and *time management related problems* [Cronbach's α = 0.79, 95% CI (0.75, 0.81)]. Example items included *I make it a habit to sleep less so that more time can be spent online* and *Going online is the first thought I have when I wake up each morning*. Participants answered on a 4-point Likert scale ranging from 1 (does not match my experience at all) to 4 (Definitely matches my experience). Higher scores indicated higher Internet addiction symptoms on all dimensions. Our choice for this scale was based on the fact that it is one of the most used instruments to assess Internet addiction symptoms, and its validity and fidelity were demonstrated in various samples and cultural contexts (60–62). The cut-off score for this scale is 63/64 points for diagnostic purposes (63). However, in the present study, we used Internet addiction as a latent variable in a SEM model, thus not the total score that would indicate clinical values based on these cut-off points. A CFA was performed, resulting in an acceptable fitted model under the orthogonal assumptions [χ^2^ = 936.78, df = 289, *p* < 0.001, TLI = 0.99, SRMR = 0.07, RMSEA = 0.07, *p* < 0.001, 90% CI (0.07, 0.08)].

A demographic scale assessed participants' age and self-reported gender. The instruments were translated using the back-translation procedure (64).

## Results

### Overview of statistical analysis

We used R (Version 4.2.3) (65) and the R-packages *dplyr* (Version 1.1.1) (66), *flextable* (Version 0.9.0) (67), *foreign* (Version 0.8.84) (68), *Hmisc* (Version 5.0.1) (69), *kableExtra* (Version 1.3.4) (70), *lavaan* (Version 0.6.15) (71), *mvtnorm* (Version 1.1.3) (72), *naniar* (Version 1.0.0) (73), *papaja* (Version 0.1.1) (74), *PerformanceAnalytics* (Version 2.0.4) (75), *psych* (Version 2.3.3) (76), *rstatix* (Version 0.7.2) (77), *sasLM* (Version 0.9.6) (78), *tinylabels* (Version 0.2.3) (74), *xts* (Version 0.13.0) (79), and *zoo* (Version 1.8.11) (80) for all our analyses.

The initial assumptions assessment was performed by descriptive univariate analysis, data screening for outliers, and missing cases analysis to verify univariate normality; the Mardia indicator (81) was computed to assess multivariate normality. A confirmatory factor analysis based on diagonally weighted least squares (82) was used to test the instruments' factorial validity and dimensional structure. Finally, the main SEM model was assessed based on robust SEM techniques and the parameters were estimated.

### Preliminary analyses

An initial descriptive analysis was performed to assess the univariate normality assumptions for the scalar variables (see Table 1). The multivariate normality assumption based on the Mardia coefficient (81) was not met, as the Mahalanobis distances from centroid coordinates were between 1.14 and 6.23. A statistically significant multivariate positively skewed (Mardia = 12.91, Skewness = 927.38, *p* < 0.001) and multivariate leptokurtic distribution (Mardia = 159.21, Kurtosis = 9.95, *p* < 0.001) were observed.

**Table 1:** Descriptive statistics for the main variables (*N* = 431).

**Variables**	***M***	**SD**	**Median**	**Min**	**Max**	**Skew (SE)**	**Kurt (SE)**
1. Rigid perfectionism	12.40	4.54	12.00	4.00	20.00	−0.08 (0.12)	−0.85 (0.23)
2. Self-critical perfectionism	17.77	6.12	18.00	6.00	30.00	−0.04 (0.12)	−0.62 (0.23)
3. Narcissistic perfectionism	13.39	5.44	13.00	6.00	30.00	0.47 (0.12)	−0.44 (0.23)
4. Alcohol use (drinking)	8.43	2.88	7.00	6.00	18.00	1.48 (0.12)	1.37 (0.23)
5. Nicotine use (smoking)	10.01	4.85	7.00	6.00	24.00	0.93 (0.12)	−0.33 (0.23)
6. Compulsion symptoms	9.89	3.72	10.00	5.00	20.00	0.47 (0.12)	−0.44 (0.23)
7. Withdrawal symptoms	10.65	3.83	11.00	5.00	20.00	0.28 (0.12)	−0.67 (0.23)
8. Tolerance symptoms	8.99	2.97	9.00	4.00	16.00	0.07 (0.12)	−0.78 (0.23)
9. Interpersonal and health problems	12.18	4.57	11.00	7.00	28.00	0.63 (0.12)	−0.49 (0.23)
10. Time management problems	9.24	3.41	9.00	5.00	19.00	0.58 (0.12)	−0.49 (0.23)
11. Subjective happiness	18.46	4.90	19.00	4.00	28.00	−0.12 (0.12)	−0.42 (0.23)

Most of Spearman's ρ correlations were statistically significant (see Table 2), with values between −0.35 and 0.78, and the correlation matrix was positively defined. Rigid perfectionism was positively associated with compulsion (ρ = 0.19, *p* < 0.001), withdrawal (ρ = 0.19, *p* < 0.001), tolerance symptoms (ρ = 0.16, *p* = 0.001), and interpersonal and health problems (ρ = 0.15, *p* = 0.002), and negatively associated with substance use—smoking (ρ = −0.10, *p* = 0.031). Self-critical perfectionism was positively associated with compulsion (ρ = 0.29, *p* < 0.001), withdrawal (ρ = 0.28, *p* < 0.001), and tolerance symptoms (ρ = 0.27, *p* < 0.001), interpersonal and health problems (ρ = 0.27, *p* < 0.001), and time management (ρ = 0.17, *p* = 0.001), and negatively associated with subjective happiness (ρ = −0.25, *p* < 0.001). Narcissistic perfectionism was positively associated with alcohol use (ρ = 0.23, *p* < 0.001), smoking (ρ = 0.14, *p* = 0.003), compulsion (ρ = 0.33, *p* < 0.001), withdrawal (ρ = 0.23, *p* < 0.001), and tolerance symptoms (ρ = 0.16, *p* = 0.001), interpersonal and health problems (ρ = 0.32, *p* < 0.001), time management (ρ = 0.28, *p* < 0.001), and negatively associated with subjective happiness (ρ = −0.11, *p* = 0.022). Next, alcohol use was positively associated with smoking (ρ = 0.50, *p* < 0.001), and negatively associated with subjective happiness (ρ = −0.25, *p* < 0.001). Smoking was also negatively associated with subjective happiness (ρ = −0.18, *p* < 0.001). Finally, all Internet addiction dimensions were negatively associated with subjective happiness (all *p*-s < 0.001).

**Table 2:** Associations between the main variables (*N* = 431).

**Variables**	**1**	**2**	**3**	**4**	**5**	**6**	**7**	**8**	**9**	**10**
1. Rigid perfectionism	–									
2. Self-critical perfectionism	0.63^**^	–								
3. Narcissistic perfectionism	0.53^**^	0.47^**^	–							
4. Alcohol use (drinking)	−0.01	0.06	0.23^**^	–						
5. Nicotine use (smoking)	−0.10^*^	−0.05	0.14^**^	0.50^**^	–					
6. Compulsion symptoms	0.19^**^	0.29^**^	0.33^**^	0.34^**^	0.25^**^	–				
7. Withdrawal symptoms	0.19^**^	0.28^**^	0.23^**^	0.24^**^	0.20^**^	0.78^**^	–			
8. Tolerance symptoms	0.16^**^	0.27^**^	0.16^**^	0.23^**^	0.16^**^	0.71^**^	0.66^**^	–		
9. Interpersonal and health problems	0.15^**^	0.27^**^	0.32^**^	0.31^**^	0.18^**^	0.75^**^	0.62^**^	0.66^**^	–	
10. Time management problems	0.04	0.17^**^	0.28^**^	0.34^**^	0.33^**^	0.67^**^	0.58^**^	0.64^**^	0.74^**^	–
11. Subjective happiness	0.05	−0.25^**^	−0.11^*^	−0.25^**^	−0.18^**^	−0.25^**^	−0.20^**^	−0.23^**^	−0.35^**^	−0.30^**^

* p < 0.05.

** p < 0.001.

**Figure 2:**
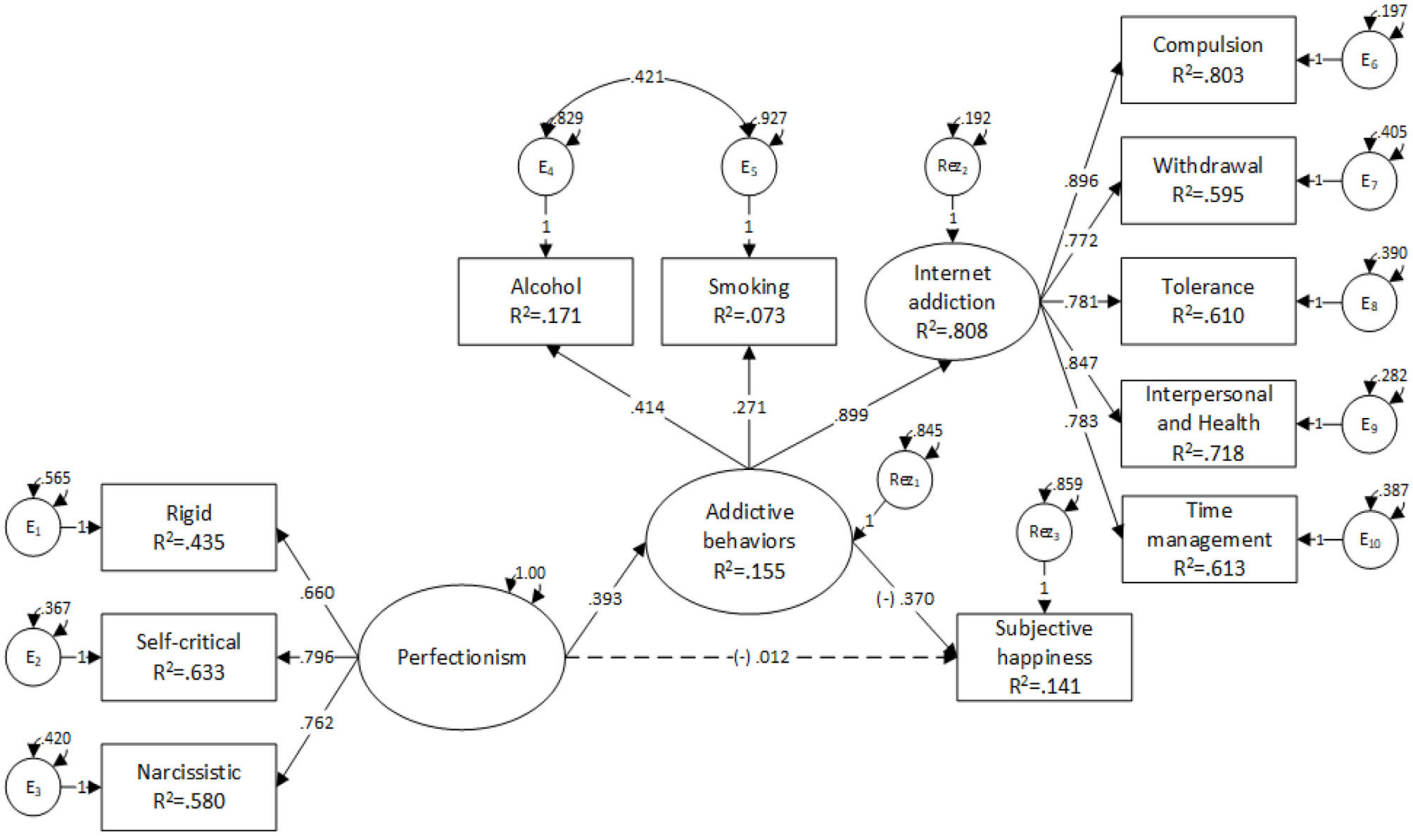
The results of the model examining the mediating role of substance use (i.e., drinking and smoking) and Internet addiction symptoms on the relationship between perfectionism and subjective happiness.

We further analyzed the main model, and convergence was acquired after 111 iterations, estimating 26 parameters based on 431 data. The modification indices suggested a model adjustment by adding a covariance between measurement errors of substance use—drinking and smoking, resulting in an over-identified model with marginal acceptable fit indices [χ^2^ = 321.40, df = 40, *p* = 0.373, CFI = 0.90, SRMR = 0.07, RMSEA = 0.07, *p* = 0.02, 90% CI (0.05, 0.08); see Figure 2].

The results suggested that addictive behaviors completely mediate the effect of perfectionism on subjective happiness. High levels of perfectionism were associated with high levels of addictive behaviors (*B* = 0.28, *z* = 5.24, *p* < 0.001, β = 0.39), and high levels of addictive behaviors were associated with low levels of subjective happiness (*B* = −0.52, *z* = −3.74, *p* < 0.001, β = −0.37). Moreover, high levels of perfectionism, mediated by addictive behaviors, were associated with low levels of subjective happiness, with perfectionism determining an indirect, statistically significant effect on subjective happiness (*B* = −0.15, *z* = −3.12, *p* < 0.001, β = −0.15). On all paths, direct and mediated, our data suggested that perfectionism was negatively associated with subjective happiness (total effect: *B* = −0.16, *z* = −2.66, *p* = 0.001, β = −0.16). However, the direct effect of perfectionism on subjective happiness was statistically insignificant (*B* = −0.01, *z* = −0.17, *p* = 0.86, β = −0.01; see Table 3).

**Table 3:** Direct and indirect effects (*N* = 431).

**Outcomes**		**Predictors**	**Estimator**	**SE**	***z***	***p*-value**	**Beta**
Addictive/Health-risk behaviors	←	Perfectionism	0.28	0.05	5.24	< 0.001	0.39
Subjective happiness	←	Perfectionism	−0.01	0.07	−0.17	0.86	−0.01
Subjective happiness	←	Addictive/Health-risk behaviors	−0.52	0.14	−3.74	< 0.001	−0.37
Subjective happiness	←	Perfectionism → Addictive behaviors	−0.15	0.05	−3.12	< 0.001	−0.15
Subjective happiness	←	Total	−0.16	0.06	−2.66	0.01	−0.16

## Discussions

Building on the Conservation of Resources Theory (25) and the Stress-Coping Model (83), the present study explored the relationship between perfectionism (rigid, self-critical, narcissistic) and subjective happiness among university students. In this relationship, we also examined the mediating roles of some specific health-risk behaviors, i.e., substance use (i.e., drinking and smoking) and Internet addiction symptoms.

We assumed that perfectionism would be positively associated with health-risk behaviors, i.e., smoking, drinking, and Internet addiction symptoms. Our results confirmed this assumption, but only in the case of narcissistic perfectionism, which was associated with all the examined health-risk behaviors. Rigid perfectionism was positively related to Internet addiction symptoms and negatively associated with smoking, but the link with drinking was insignificant. Furthermore, self-critical perfectionism was positively related to Internet addiction symptoms, but the link between drinking and smoking was insignificant. These results highlight some specific aspects of the negative outcomes regarding health-risk behaviors concerning narcissistic perfectionism. As previous research highlighted, narcissistic perfectionists are usually highly critical; they can be demanding and arrogant due to feelings of entitlement (38). Due to the conflicts generated by these behaviors, our results confirm the assumption that they might be more prone to engage in compensatory behaviors, such as drinking, smoking, and addictive Internet use, to manage the related emotional negativity.

Previous studies also suggested that individuals high in narcissistic perfectionism are also highly focused on being and seeming perfect (23). Thus, narcissistic perfectionists are vulnerable when confronted with the reality that they are not perfect and never will be flawless via the trials and tribulations of everyday life. When their private insecurities become public, the drive to seem flawless is coupled with a sense of hopelessness regarding recovery from public shame and embarrassment. Consequently, they might engage in addictive Internet use to build, maintain, manage, and protect their *perfect* public image. Furthermore, this effort might increase their proneness for other health-risk behaviors, such as drinking and smoking (84), and the present research results also underlined this significant positive association. At the same time, this striving for perfection comes with the price of addiction and risky behaviors, leading to lower subjective happiness (23, 85).

Next, we assumed that health-risk behaviors (i.e., drinking, smoking, and using the Internet addictively) would be related to lower subjective happiness. Our assumption was confirmed, with negative associations between all the examined risky behaviors and subjective happiness. Moreover, it seems that students who smoke and drink might be more prone to engage in addictive Internet use. These outcomes align with previous findings suggesting that health-risk behaviors (e.g., drinking) are generally negatively associated with subjective happiness among youth (86, 87). More importantly, these results highlight the need to assess and intervene when health-risk behaviors, such as drinking, smoking, and Internet addictive behavioral patterns emerge among students due to the various adverse negative consequences on their mental and physical health.

Finally, our study's most important result highlights that health-risk behaviors (smoking, drinking, and addictive Internet use) fully mediated the link between perfectionism and subjective happiness. There are both theoretical and practical considerations to discuss concerning these specific results. On the one hand, the present findings add to the scarce literature examining the multidimensional facets of perfectionism and their indirect link with subjective happiness. On the other hand, the present results highlight the specific maladaptive outcomes of narcissistic perfectionism and the need to address such cognitive and behavioral patterns due to (1) the risk of developing Internet addiction and engaging in smoking and drinking to cope with the negative emotions fueled by not meeting perfectionist standards, and (2) their significant indirect effect on subjective happiness, which further translates into a higher risk for developing psychological distress.

Though we did not use cut-off cores that would indicate whether our sample scored in the clinical or subclinical groups, descriptive statistics of the factors indicated a rather subclinical sample and some recommendations for preventive measures can also be considered, given the increasing prevalence of Internet-related addictions among youth (88). Also, recommendations for preventive measures in subclinical samples are also useful to manage the associated risk factors (in this case, perfectionism), to prevent developing health risk behavioral patterns into severe addictions or substance abuse disorders to enhance resilience and lower vulnerability. More specifically, the findings of the present study might bring important insights for interventions aimed at (1) reducing health-risk behaviors among students and (2) increasing subjective happiness by addressing the significant direct effect of perfectionism (on smoking, drinking, and addictive Internet use), as well as its indirect influence (on subjective happiness). In order to do that, parents, teachers, and students must be familiarized with the troubling signs of high perfectionism and its maladaptive function without ignoring its beneficial effects. We know from previous studies that adaptive perfectionism might contribute, for instance, to reducing academic procrastination (89) and enhancing creativity (90). Thus, a clear distinction between adaptive and maladaptive facets of perfectionism should be at the core of such interventions. Some possible ways to spot maladaptive perfectionism, especially narcissistic perfectionism, might include private conversations—face-to-face or online, group exercises, and formal and informal teacher-student contexts. The signs that might reflect a student's narcissistic perfectionism could include, for instance, the inability to get satisfaction from personal, professional, or leisurely pursuits because of their lofty expectations of themselves and others. Furthermore, anger and sadness stemming from unmet expectations of entitlement might as well contribute to the prevalence of depressive symptoms, translated into online and offline behaviors and speech, daily interactions with students and teachers, and even their GPA scores. Finally, similar to the cases of therapists treating individuals with high narcissistic perfectionism, teachers might feel worthless and inadequate, impatient and exploited, as common responses to such students' grandiosity and fragility. Thus, such signs should be spotted among both students and teachers, and further addressed in interventions aimed at reducing the negative effects of maladaptive perfectionism.

Furthermore, since drinking, smoking, and addictive Internet use might be used as coping mechanisms to deal with the frustration and negative emotions, in general (due to not meeting perfectionist standards), prevention and intervention campaigns among students might as well be effective in dealing with the negative outcomes of perfectionism, and its ramification on students' physical and mental wellbeing. Some examples in this regard are described in previous studies which highlight the important role of self-compassion when addressing maladaptive perfectionism (91). Also, multi-level ecological strategies are also recommended when discussing university-based interventions (instead of using a single level approach) aimed at reducing addictive behaviors among students (92).

However, there are also a series of limitations to be considered in the present study. First, we used convenience sampling, lowering the generalizability of the present findings. Second, we relied on self-reported measurements, which may have been skewed toward positive responses. The suggested research model may benefit from experimental analysis in the future (for instance, when measuring perfectionism) (93). Also, the scale that we used to measure health-risk behaviors was originally designed for younger samples. Though our sample was formed by young adults, i.e., students, and in Romania—according to the official statistics, most of them (i.e., more than 50%) live with their parents (94), future studies might benefit from using alternative measurements (designed for students) to address this limitation. Next, our methodology was cross-sectional, which prevented us from establishing a causal connection; future longitudinal studies might be employed to overcome this shortcoming. In addition, the proposed factors are not the only ones that influence the relationship between the proposed variables; additional variables may also be key predictors and moderators of the relationship between students' perfectionism and subjective happiness, such as autonomy, environmental mastery, and purpose in life (95), personality factors and resilience (96), intellectual giftedness (97), or achievement goals (98). Also, pre-existing clinical conditions of the participants might also account for variability in the present findings (e.g., previously diagnosed personality disorders), which might be controlled in further studies.

## Conclusion

The present findings highlight the significant negative indirect effect of perfectionism on students' subjective happiness through the mediating effect of health-risk behaviors, i.e., smoking, drinking, and addictive Internet use. Moreover, the current results highlight the maladaptive function of narcissistic perfectionism and its significant link with alcohol and nicotine use and maladaptive Internet use. Though in need of further research, this study might have a significant theoretical and practical contribution in addressing the negative outcomes of students' perfectionism on their subjective wellbeing, as well as their physical health.

## Data availability statement

The raw data supporting the conclusions of this article will be made available by the authors, without undue reservation.

## Ethics statement

The studies involving humans were approved by Alexandru Ioan Cuza University, Faculty of Psychology and Education Sciences. The studies were conducted in accordance with the local legislation and institutional requirements. The participants provided their written informed consent to participate in this study.

## Author contributions

All authors listed have made a substantial, direct, and intellectual contribution to the work and approved it for publication.
